# The dual threat of hypervirulent and multidrug-resistant *Klebsiella pneumoniae*: pathogenesis and prospects for intervention

**DOI:** 10.3389/fmicb.2026.1837929

**Published:** 2026-06-23

**Authors:** Sayani Roy, Kamalina Choudhury, Uelinton Manoel Pinto, Vijay K. Singh

**Affiliations:** 1 Infectious Disease and Antimicrobial Resistance Laboratory, Division of Life Sciences, Institute of Advanced Study in Science and Technology (IASST), Guwahati, Assam, India; 2 Academy of Scientific and Innovative Research (AcSIR), Ghaziabad, India; 3 Laboratory of Food Microbiology, Food Research Center (FoRC), Department of Food and Experimental Nutrition, School of Pharmaceutical Sciences, University of São Paulo (USP), São Paulo, Brazil

**Keywords:** anti-virulence, host-pathogen interactions, hypervirulent *Klebsiella pneumoniae*, multidrug resistance, quorum sensing-inhibition, virulence plasmids

## Abstract

*Klebsiella pneumoniae* (Kp) has emerged as a major public health concern and is listed by the World Health Organization (WHO) as a critical-priority pathogen. Its clinical relevance is amplified by the rise of hypervirulent *Klebsiella pneumoniae* (hvKp), which combines enhanced pathogenicity with an alarming potential to acquire multidrug resistance (MDR). Unlike classical *K. pneumoniae* (cKp), hvKp causes severe community-acquired infections, including pyogenic liver abscess, meningitis, septic endophthalmitis, and necrotising fasciitis, leading to significant morbidity and mortality. Since the first reports from Taiwan in the 1980s, hvKp has spread globally, with convergent MDR-hvKp strains increasingly documented in Asia, North America, and Europe, posing urgent therapeutic challenges. The virulence of hvKp is largely driven by plasmid-encoded factors, including hypermucoid regulators (rmpA/rmpA2), siderophores such as aerobactin and salmochelin, and heavy-metal resistance genes. While phenotypic assays like the string test have historically been used, they lack specificity, and molecular biomarkers such as *rmpA*, *rmpA2*, *iroB*, *iucA*, and *peg-344* remain inconsistently distributed across isolates. This complicates accurate differentiation of hvKp from cKp and underscores the pressing need for robust, standardized biomarker panels. This article reviews current knowledge on hvKp pathogenesis with a focus on gut colonization, immune evasion, and niche-specific regulation of virulence factors. We also examine the role of quorum sensing in fine-tuning hvKp virulence and highlight emerging anti-virulence strategies as promising alternatives to traditional antibiotics. By bridging mechanistic insights with translational approaches, this review aims to inform the development of effective diagnostic and therapeutic strategies against hvKp.

## Introduction

*Klebsiella pneumoniae*, a Gram-negative, non-motile, opportunistic pathogen, is responsible for various nosocomial illnesses. It has been ranked as the fourth deadliest pathogen globally and designated as a critical priority pathogen by the World Health Organization (WHO) (Ikuta et al., 2022). This “superbug” has the potential to readily acquire antibiotic resistance and virulence determinants. Over the years, *K. pneumoniae* has evolved into two pathotypes: classical *K. pneumoniae* (cKp) and hypervirulent *K. pneumoniae* (hvKp). While cKp is associated with nosocomial infections in immunocompromised patients, hvKp is responsible for severe metastatic and invasive infections transmitted primarily in community settings (Ng and Frazee, 2015; Zhao et al., 2021). This invasive nature of hvKp is attributed to its enhanced virulence arsenal, viz., the hypermucoviscous phenotype, overproduction of siderophores, and resistance to phagocytosis, which enable it to breach host barriers more effectively than cKp. HvKp infection mainly manifests as liver abscess, including septic endophthalmitis, meningitis and necrotising fasciitis (Zhao et al., 2021).

Since its first report from Taiwan in 1986 (Liu et al., 1986), it has been sporadically spreading in Asian countries, including China, Japan, South Korea and India over the years (Gan et al., 2025). Initially, the hvKp strains were drug sensitive. However, with time, alarmingly high mortality rates for infected patients have been reported due to the rising incidence of convergent multidrug-resistant hvKp (MDR-hvKp) (Mukherjee et al., 2020; Shankar et al., 2020; Wyres et al., 2020b). The convergence of antibiotic resistance and virulence determinants in this superbug led to poor treatment outcomes and limited therapeutic options.

Often community-acquired, hvKps inhabit various host niches depending upon the stage of infection. In many cases, they colonize the gastrointestinal tract asymptomatically, as demonstrated by reports showing that hvKp is detected in the gastrointestinal tracts of healthy individuals in China and Malaysia (Russo and Marr, 2019; Yang et al., 2022). Additionally, the close genetic relatedness between hvKps from human feces and those from liver abscesses supports this hypothesis (Chung et al., 2012; Fung et al., 2012). Liver abscess due to hvKp has also been reported from the US, Canada and several European countries, predominantly linked to pathogen carriage in migrants originating from Asian countries (Raro et al., 2023; Siu et al., 2012).

The hvKp harbors a large virulence plasmid, typically of the IncFIBK type, that carries multiple virulence genes absent or present at low prevalence in cKp. Virulence gene clusters, such as regulator of hypermucoid phenotype in hvKp (*rmpA/rmpA2*), siderophore production genes such as aerobactin (*iucABCD*, *iutA*), and salmochelin (*iroBCDN*) are carried in by this virulence plasmid (Russo and Marr, 2019). The importance of the virulence plasmid and the contribution of individual virulence determinants during systemic infection have been documented in various studies (Chu et al., 2023; Nassif and Sansonetti, 1986; Tang et al., 2025). A common complication caused by hvKp is bacteremia, besides infecting multiple sites, such as the eyes, lungs, liver, kidneys, spleen, fascia, and the central nervous system of a host. Also, hvKp is capable of causing invasive as well as metastatic infections in apparently healthy individuals, which are unusual for cKp infections. Given these complications, and lack of a universally accepted criteria for distinguishing hvKp from cKp, a systematic evaluation of current diagnostics and classification approaches is essential.

Recently, there has been a growing focus on elucidating the intricate relationship between hvKp and its host environment. The ability of hvKp to colonize the gut asymptomatically, evade the host immune system, and induce invasive symptoms illustrates a precise relationship between the virulence genes and the regulatory systems that control them. Its niche-specific adaptation and the expression of distinct sets of virulence genes at different stages of infection are crucial for controlling and managing this pathogen. This review, therefore, aims to compile the current understanding of the mechanisms by which hvKp colonize the gut, including the factors responsible for their intestinal persistence. The niche-specific roles of virulence determinants involved in colonization and invasion, and the roles of quorum sensing regulators in the virulence regulation of hvKp are also explored in this review. Further, recent antivirulence strategies aimed at mitigating the burden of hvKp infection have also been summarized. Overall, this review offers an integrated perspective on the virulence of hvKp, bridging basic pathogenesis with emerging antivirulence strategies, which will be helpful in future research and therapeutic strategies.

## Diagnostics and classification challenges

The accurate diagnosis of hvKp for clinical management remains a major challenge for clinicians. Although significant progress has been made in understanding the molecular basis of virulence, inconsistencies in phenotypic assays and genotypic biomarkers complicate their identification. Thus, the development of a robust and standardized panel of biomarkers is essential for distinguishing hvKp from cKp. Since hvKp was first identified in Taiwan, it has been characterized and differentiated from cKp primarily based on its hypermucoviscous phenotype, which is commonly assessed using the string test. Due to its simplicity and low cost, the string test was earlier considered the gold standard for identifying hvKp based on its hypermucoviscous phenotype, where formation of a viscous loop greater than 5 mm indicated a positive result (Fang et al., 2004; Liu et al., 1986). However, later studies found that not all hvKps produce hypermucoviscosity, and some cKp isolates can also produce this hypermucoviscous phenotype (Yu et al., 2008) which made the string test inaccurate for identifying hvKp. Similarly, sedimentation resistance and uronic acid assays for capsule quantification can yield false negatives due to growth conditions that modulate the hypermucoviscous phenotype and hypercapsule production. Alternatively, *Galleria mellonella* infection model has also been utilized for determining the *in vitro* neutrophil-mediated bactericidal assay to determine the pathogenicity of hvKp (Dai and Hu, 2022; Russo et al., 2021). However, this method also cannot accurately differentiate between hvKp and cKp as cKp has been shown to possess resistance to neutrophil-mediated killing (Russo and MacDonald, 2020). The current standard for determining the pathogenicity of hvKp *in vivo* is the murine infection model (Tang et al., 2025). However, ethical concerns, high cost, and operational complexity make it impractical for clinical studies and for the surveillance of large collections of strains.

Recent evidences support the presence of five hvKp virulence-associated plasmid genes- *rmpA, rmpA2, iucA, iroB*, and *peg 344*- which provide the most reliable method of identifying hvKp (Klaper et al., 2021; Neumann et al., 2023; Rao and Fernandes, 2025; Russo et al., 2018). Several studies have reported hvKps, presumptively based on the presence of only a subset of defining genotypic markers- *rmpA, rmpA2, iucA, iroB* and *peg 344* and/or partial segments of the canonical virulence plasmid of hvKp (Gu et al., 2018; Hu et al., 2023a; Lai et al., 2003; Yang et al., 2020). Whole genome sequencing is also one of the approaches for definitive identification of the virulence genes of hvKp (Pustam et al., 2024; Shankar et al., 2018; Tang et al., 2025). Despite its high resolution, the routine clinical use of WGS is limited by its high cost, specialized personnel and lack of standardized genomic criteria of hvKp. Although several assays have been developed to identify hvKps, they have been documented in isolated studies and lack reproducibility across independent investigations (Fernández Vecilla et al., 2025; Yan et al., 2023; Zhu et al., 2025). These inherent limitations underscore the need for integrated and standardized approaches to ensure accurate identification.

## Colonization dynamics and niche adaptation

Epidemiological research suggests that the most common way that hvKp is carried in people, especially in Asian populations, is by gastrointestinal colonization. According to surveys conducted in South Korea, China, Taiwan, Hong Kong, Singapore, and Vietnam, stool samples from 4-6% of healthy persons contain ST23/K1 hvKp (Chung et al., 2012; Fung et al., 2012; Russo and Marr, 2019; Yang et al., 2022), indicating the intestine as a reservoir of this concerning public health threat. Notably, ST23 first emerged in Taiwan in the mid-1980s as a hypervirulent *Klebsiella pneumoniae* lineage causing metastatic infections and pyogenic liver abscess (Russo and Marr, 2019). It harbored all the virulence markers of hvKp, including the K1 capsular polysaccharide, rmpA/rmpA2 regulators, and iron-acquisition systems (aerobactin, salmochelin) on the virulence plasmid (Khrulnova et al., 2022; Lazăr et al., 2026; Sohrabi et al., 2022). Collectively, these findings suggest that the acquisition of hvKp probably occurs through a combination of fecal-oral pathways, environmental sources, and community transmission. Although hvKp is still mostly community-associated, exposure to healthcare facilities and international travel, particularly to endemic areas, appears to increase the risk of colonization (Baronos et al., 2025; Lee et al., 2016; Russo and Marr, 2019).

Murine models provide complementary insights into the mucosal behavior of hvKp, revealing distinct tissue tropisms and colonization dynamics. According to results obtained from murine infection models, unlike cKp, whose initial site for colonization and replication is the nasal cavity or nasopharynx, hvKp replicates in the lungs and mediastinal adipose tissue, with nasal tropism playing a less significant role (Teo et al., 2024). HvKp colonizes the gut, establishing a high-load colonization within the ileum of the small intestine (Caballero et al., 2015; Hennequin and Forestier, 2009). However, the study of Teo et al. (2024) and Young et al. (2020) in murine infection model suggests that this colonization is preceded by direct contact of hvKp with the esophageal mucosa, indicating that the environmental signal along with direct mucosal interaction in the upper gastrointestinal tract may stimulate hvKp to enhance niche-specific virulence factors that transiently overcome colonization resistance. A likely mechanism facilitating this adaptation is the synergistic effect of microcin E492 and colibactin encoded by the genomic island, GIE492 and the integrative conjugative element K10 (ICEKp10), respectively, harbored by the hvKp strains. These factors cooperatively diminish the neighboring gut commensals, creating a more permissive niche for hvKp persistence facilitating successful intestinal seeding and potential translocation (Tan et al., 2024). However, these observations are largely derived from murine infection models, and it remains unclear whether they translate to human infections. Nevertheless, such data may serve as a framework for comparative analyses of clinical manifestation and outcomes in hvKp and cKp.

## Pathogenesis and tissue tropism

**Figure 1:**
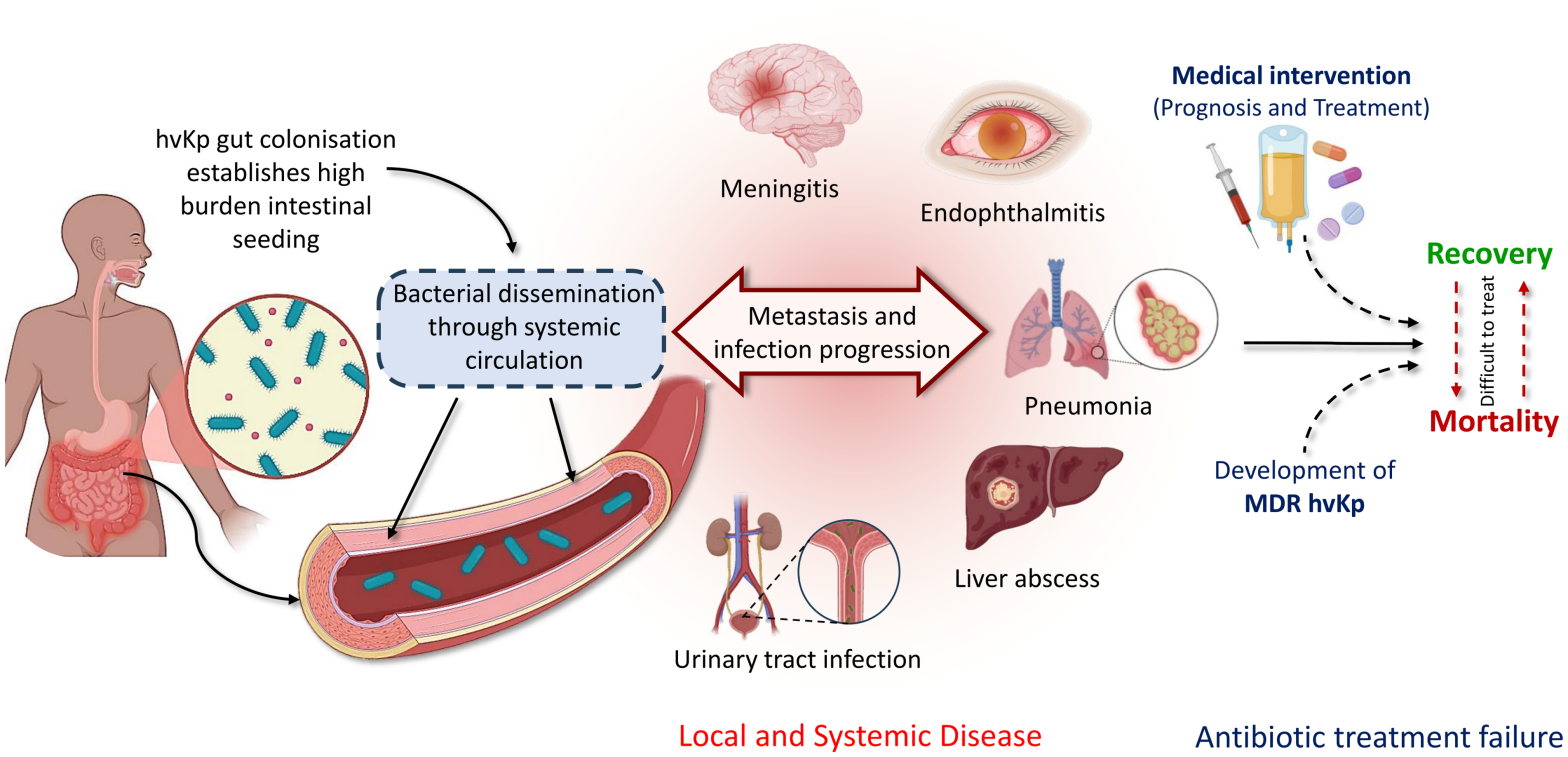
Pathogenesis of hypervirulent *Klebsiella pneumoniae* (hvKp). Gut colonization by hvKp establishes a high intestinal bacterial burden, enabling translocation into the bloodstream and systemic dissemination causing both local and systemic infections. Clinical outcomes depend on timely and effective medical intervention. However, the emergence of multidrug-resistant hvKp (MDR hvKp) leads to treatment failure and an increase in mortality rate. Created with BioRender.com.

The invasive and disseminating nature of hvKp has exacerbated the chances of high morbidity and mortality in infected patients (Hwang et al., 2020; Lan et al., 2020). Pyogenic liver abscess was the first reported symptom of hvKp infection (Lederman and Crum, 2005). Formation of pyogenic liver abscess is a coordinated process that combines host vulnerability and strong bacterial virulence and is typically associated with the host gut colonization by hvKp, establishing a high burden of intestinal seeding (Fang et al., 2007; Psonis et al., 2022). The bacteria translocate across the intestinal barrier and enter the systemic circulation, leading to metastatic spread. Poor prognosis and emergence of multidrug-resistant hvKp strains contribute significantly to increased mortality (Figure 1; Chen et al., 2025). Immunocompromised diabetic mice are more vulnerable to hvKp invasion and result in more severe lethality than in non-diabetic mice. These findings are consistent with clinical observations indicating that diabetes mellitus is a major risk factor for invasive hvKp infections in humans (Lin et al., 2017; Tang et al., 2023; Thimmappa et al., 2023; Wang L. et al., 2023; Wu and Tsai, 2005). The bacterium from the gut traverses the intestinal barrier and spreads hematogenously to the liver. HvKps may also establish primary infections in the liver via bloodstream dissemination, supported by a high bacterial load in the systemic circulation (Psonis et al., 2022; Serban et al., 2021).

Upon reaching the liver, an array of virulence determinants such as *rmpA, rmpA2* and *wzy* regulating the hypercapsule are deployed to initiate the infection. The production of hypercapsule makes the bacterium resistant to phagocytosis, complement, and antimicrobial peptides (Hu et al., 2023b). HvKp serotypes K1 and K2, are particularly associated with invasive infections, with K1 hvKp strains often linked to pyogenic liver abscesses (Shon et al., 2013). Also, experimental evidence from a murine model has demonstrated that antibiotic-induced dysbiosis disrupts an axis called the indole propionic acid-interleukin-22-Reg3b axis, which is crucial for intestinal defence. This disruption makes it easier for hvKp to translocate to the liver, resulting in the formation of an abscess (You et al., 2024). The niche adaptability of hvKp is further strengthened by the allantoin metabolism gene *allS* and the plasmid-encoded metabolite transporter *peg-344,* which enable the bacterium to proliferate in the liver (Gan et al., 2022).

As the infection progresses, the hypercapsule augments the TNF-*α* production while simultaneously inhibiting the release of pro-inflammatory cytokines, including IL-1β. This helps the hvKp modulate the local immune response, thereby facilitating immune evasion and tissue damage (Hu et al., 2023b). In gas-forming pyogenic liver abscesses, primarily seen in diabetic individuals, hvKp metabolizes glucose via mixed-acid fermentation pathways, yielding hydrogen and carbon dioxide. The buildup of gas in abscesses is linked to worse clinical outcomes and is a sign of severe hvKp-driven liver disease (Lee et al., 2004). ST23, the unique sequence type identified in liver abscess isolates, is closely linked to hypervirulent phenotype and has spread globally. Moreover, as reported in a previous study, *K. pneumoniae* isolates collected from liver abscesses harbored more virulence genes than those collected from patients with pneumonia. Also, the prevalence of type VI secretion system (T6SS) and salmochelin in isolates sourced from pyogenic liver abscesses is higher than that of the intestinal-colonizing hvKp strains (Gan et al., 2022; Hsieh et al., 2019). This suggests that hvKp encodes additional virulence factors for immune evasion, which are critical for establishing infection and forming a pyogenic liver abscess.

A recent epigenetic study has revealed that the virulence loci in hvKp, including those encoding the iron acquisition systems and CPS are significantly enriched in hypermethylated GATC and CCWGG motifs (Ghosh et al., 2024). This study suggests a potential regulatory role of DNA methylation which requires further experimental validation to assess its contribution to the virulence and pathogenicity of hvKp. Thus, multiple regulatory strategies confer hvKp a survival advantage during host invasion, enabling rapid adaptation and immune evasion, thereby making them more virulent than their classical counterparts.

## Genetic architecture of hvKp virulence

**Figure 2:**
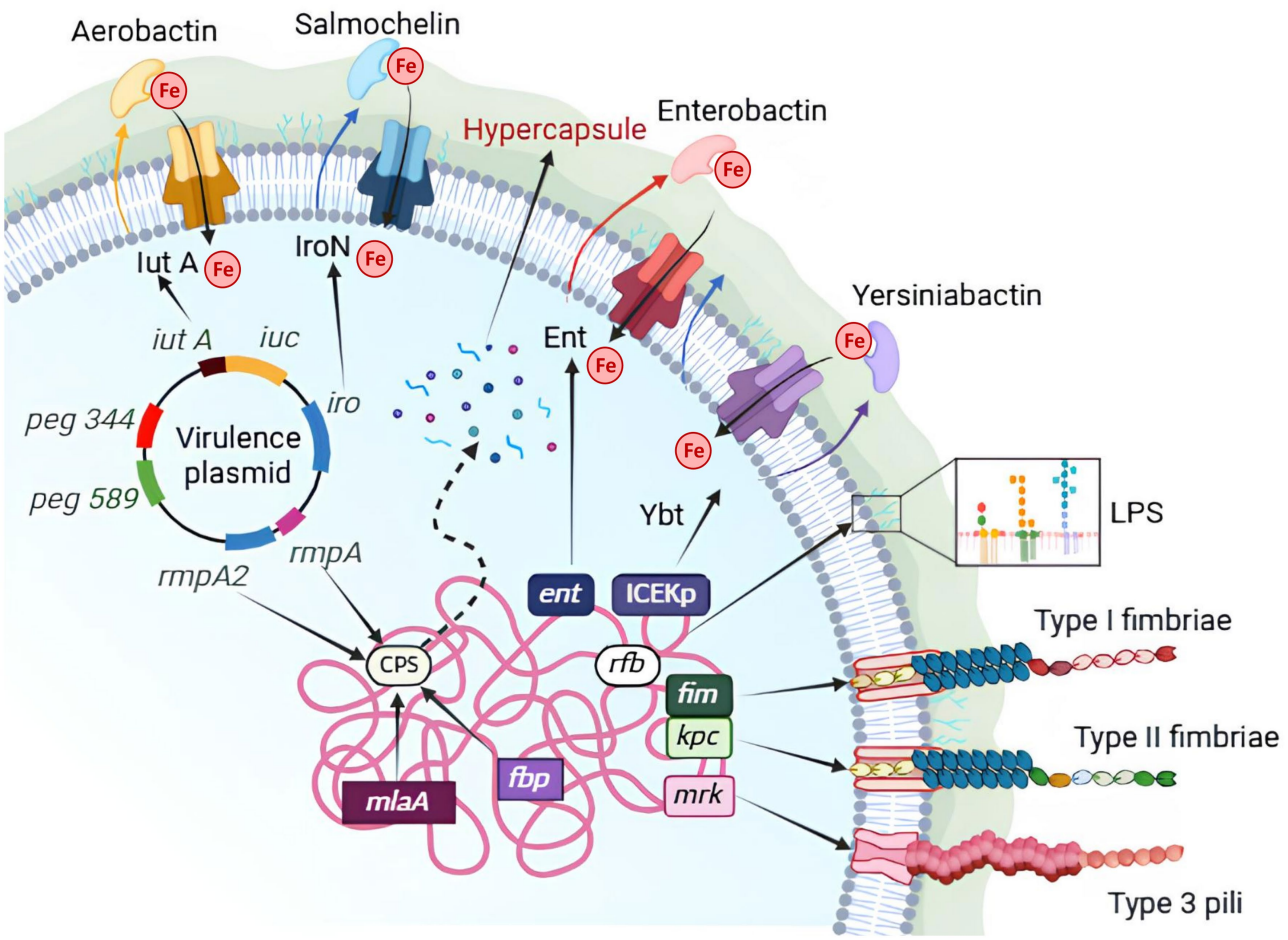
Schematic overview of key genetic elements associated with hvKp. The image illustrates chromosomal and plasmid-encoded virulence determinants, including siderophore systems (*iuc, iutA, iro*), regulators of mucoid phenotype (*rmpA, rmpA2*), and additional plasmid-borne genes (*peg-344*, *peg-589*). Chromosomal factors such as *fim, mrk, ent, rfb, CPS, ICEKp, mlaA*, and *fbp* contribute to adhesion, iron acquisition, and capsule synthesis, promoting enhanced virulence and persistence. The figure highlights the complex genetic basis underlying hvKp pathogenicity. Created with BioRender.com.

The virulence of hvKp strains is primarily driven by a large plasmid encoding multiple virulence loci, distinguishing these strains from their classical counterparts. The virulence plasmid, about 200 kb-300 kb long, encodes genes for siderophores (aerobactin *iucABCD*/*iutA* and salmochelin *iroBCDN*), regulators of mucoid phenotype (*rmpA* and *rmpA2*), *peg-344*, and, more recently reported, *peg-589* (Tang et al., 2025; Wyres et al., 2020b; Figure 2). These genes are crucial for their survival and growth in iron-limited host environments making them invasive and significantly more pathogenic than cKp (Russo and Marr, 2019; Wu et al., 2022). Initially, sequencing of hvKp strains identified two large, highly similar virulence plasmids, pK2044 (224,152 bp) and pLVPK (219,385 bp), which carry the virulence markers of hvKp. Experimental plasmid curing eliminates hypervirulence phenotypes, highlighting their necessity, while horizontal transfer-frequently through conjugative mechanisms involving IS26 elements-has propagated these elements to cKp lineages, resulting in concerning multidrug-resistant hvKp (MDR-hvKp) hybrids (Chen et al., 2004; Tang et al., 2010; Wu et al., 2009). Two smaller virulence plasmids, one 121-kb in size, contained *iuc*, *iro*, and *rmpA*, whereas the second, a 90-kb plasmid, contained *rmpA2* in the hvKp strain Kp52.145 has also been reported (Lery et al., 2014). Most hvKp strains isolated from liver abscess or community-acquired pneumonia harbor pLVPK-like plasmids encoding the core loci namely, *iuc, iro, rmpA*, and *rmpA2*, though some exhibit deletions in flanking regions (Struve et al., 2015). Similarly, Lam et al., analyzed 94 CG23 group of hvKp strains and detected pK2044-like virulence plasmids where *iro* was conserved in all 94 plasmids, *iuc, rmpA*, and *rmpA2* showed near complete presence (92/94), highlighting modest variability within this dominant plasmid backbone (Lam et al., 2018).

Complementing these plasmid-encoded determinants, chromosomal multilocus sequence typing (MLST) profiles distinguish hvKp clonal lineages by integrating virulence plasmids with lineage-specific genetic variants. Strains belonging to the ST-23 sequence type with K1 serotype are the dominant lineage causing hypervirulent infections, particularly causing pyogenic liver abscess (Struve et al., 2015). These strains have unique islands such as KPHPI208, which encodes yersiniabactin (ybt), colibactin (pks), and microcin E492, while ST65, ST86, and ST66, belonging to K2 serotype have regional prevalence and ST11 emerging as extensively drug-resistant hvKp hybrids (He et al., 2022; Li et al., 2025).

Recent studies have also highlighted the chromosomal integration of virulence plasmids or gene fragments in hvKp, enhancing the genetic stability of these virulence factors. A virulence plasmid harbored by hvKp isolate ST420, containing *rmpA/rmpA2, iucABCDiutA, iroBCDN,* and *peg-344* was found to be integrated into the chromosome via ISKpn74 flanked by transposases with identical direct repeats (Eger et al., 2021). A similar mechanism was seen in ST11 carbapenem-resistant hvKp where a 107 kb chromosomal fragment containing plasmid-derived *rmpA2* and *iucABCDiutA* and was flanked by IS26 elements (Yang et al., 2020).

Some important differences between hvKp and cKp are shown in Table 1.

**Table 1:** Differences between hypervirulent *K. pneumoniae* and classical *K. pneumoniae.*

Hypervirulent *K. pneumoniae*	Classical *K. pneumoniae*	References
Predominantly community acquired and frequently infects healthy individuals	Responsible mainly for nosocomial and opportunistic infections typically infecting immunocompromised individuals	Kamau et al. (2022), Pendleton et al. (2013), Russo et al. (2021), Russo and Marr (2019), and Wyres et al. (2020a)
Responsible for metastatic and invasive infection	Responsible for localized and non-invasive infection	Russo et al. (2021), Russo and Marr (2019), and Shon et al. (2013)
Typically harbors a virulence plasmid containing the characteristic virulence genes of hvKp (*rmpA,rmpA2, iucA, iroB and peg344*)	Absence or rare presence of a virulence plasmid	Huang et al. (2023) and Lim et al. (2025)
Produces high level of siderophore, particularly aerobactin and salmochelin	Primarily produces enterobactin with lower siderophore activity	Abbas et al. (2024), Liao et al. (2024), Pu et al. (2023), and Wu et al. (2022)
Commonly associated with K1/K2 serotype	Exhibits diverse capsular serotypes; K1 and K2 serotypes are less prevalent	Al-Busaidi et al. (2024) and Shafaie et al. (2025)
Demonstrated increased virulence in mouse infection model	Comparatively lesser virulence in mouse infection models	Gan et al. (2022) and Russo et al. (2021)
The biofilm formation ability is stronger.	Weaker biofilm-forming ability compared to hvKp	Wen et al. (2024) and Wu et al. (2011)
HvKp strains with hypermucoviscosity are tolerant to triclosan, sodium hypochlorite, and benzalkonium bromide	cKp are non-tolerant to triclosan, sodium hypochlorite, and benzalkonium bromide	Wei et al. (2024)
The initial sites of colonization and replication of hvKp are the lungs and mediastinal adipose tissue.	The initial site for colonization and replication of cKp is the nasal cavity (nasopharynx).	Joseph et al. (2021) and Teo et al. (2024)
Prevalence of the ferric uptake system (*kfuABC*) is higher in hvKp	Prevalence of the ferric uptake system (*kfuABC*) is less in cKp	Holt et al. (2015)

**Figure 3:**
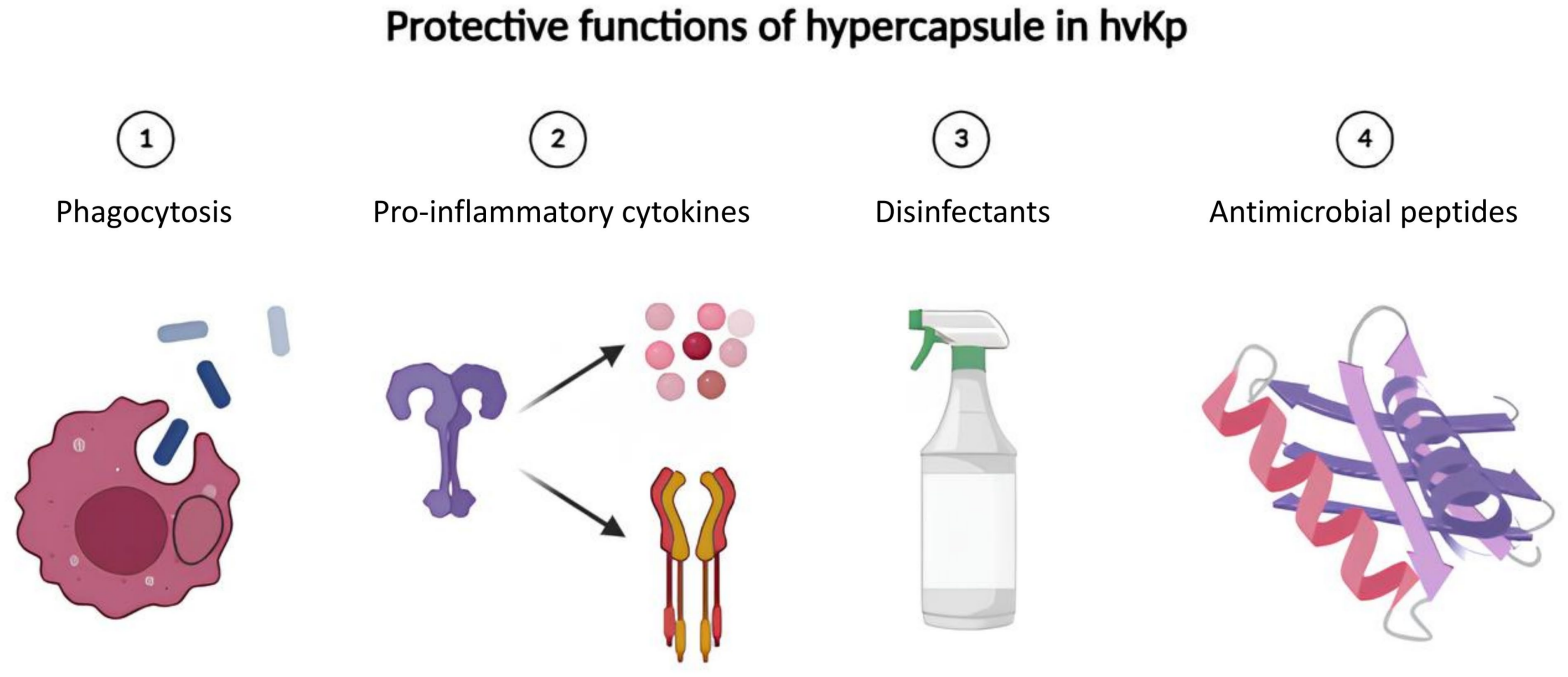
Schematic representation of hvKp resistance to disinfectants, antimicrobial peptides, phagocytosis, and pro-inflammatory cytokines, primarily mediated by its hypercapsule. The hypercapsule acts as a physical and functional barrier, protecting hvKp against host immune defenses and environmental stressors, thereby enhancing its survival and virulence. Created with BioRender.com.

The capsular polysaccharide (CPS) of *K. pneumoniae* plays a pivotal role in determining the hypervirulent phenotype by enabling evasion of host immune responses and facilitating tissue invasion. Hypervirulent strains exhibit a hallmark hypermucoid appearance due to overproduction of capsule, which renders them highly resistant to phagocytosis and serum-mediated killing (Tsubaki et al., 2026; Yeh et al., 2007). Due to this hypermucoidity, the hvKps are also resistant to broad-spectrum antimicrobial disinfectants, triclosan, sodium hypochlorite, and benzalkonium bromide (Wei et al., 2024; Figure 3). Central to this process of overproduction of capsule is the plasmid-encoded *rmp* locus, notably the *rmpADC* operon (Lai et al., 2003; Xu et al., 2024; Yang et al., 2023). While *rmpA* and *rmpA2* have long been associated with capsule regulation, recent findings reveal that *rmpD* and *rmpC* act in concert to regulate capsule production. While *rmpD* boosts the production of the hypermucoid phenotype, *rmpC* adjusts capsular biosynthesis (Salisbury et al., 2026; Walker et al., 2019, 2020).

However, hypermucoviscosity in hvKp requires capsular polysaccharides, along with other biochemical factors and the cell’s metabolic status (Lai et al., 2003; Mikei et al., 2021). Both chromosomally encoded and plasmid-borne genes intricately regulate capsule synthesis in hvKp. The chromosomal *cps* cluster gene *wcaJ*, which encodes the glycosyltransferase, is important for capsule formation (Devanga Ragupathi et al., 2020; Wang W. et al., 2023). But *wcaJ* variants are serotype-specific, limiting their functionality across strains. RmpA, a conserved regulator, enhances the promoter activity within this cps cluster that further modulates *cps* expression (Cheng et al., 2010; Wang W. et al., 2023). RmpD was shown to interact with Wzc (a conserved capsule biosynthesis protein), producing longer, more uniform chains of capsular polysaccharide, a feature linked to enhanced virulence (Ovchinnikova et al., 2023).

Capsule biosynthesis has also been reported to be influenced by the iron availability. IroP in an hvKp strain SGH10, a regulatory protein embedded within the salmochelin operon, plays a role in modulating the process of capsule formation (Choby et al., 2019; Chu et al., 2023). Under iron scarcity, IroP suppresses the expression of type 3 fimbriae on the chromosome while enhancing capsule biosynthesis. But in the presence of iron abundance, *iroP* is repressed by the iron-bound Fur, which relieves the type 3 fimbriae suppression, decreasing mucoviscosity and enabling adhesion. This modulation, mediated through coordinated plasmid-chromosome interplay, enables hvKp to enhance its virulence strategies, shifting between immune evasion and tissue colonization (Chu et al., 2023). However, the precise role of *iroP* in capsule regulation requires further investigation for its validation across hvKp lineages.

Besides these, hypermucoviscosity is also regulated by *mlaA* (an outer membrane lipoprotein implicated in retrograde phospholipid trafficking) and *fbp* (which encodes fructose-1,6-bisphosphatase), located outside the *cps* cluster. While *fbp* and *mlaA*, have different roles in metabolic pathways, their ablation significantly reduces the hypermucoviscosity (Wu et al., 2024). The outer membrane integrity of hvKp might be influenced by MlaA’s retrograde phospholipid trafficking while *fbp-*mediated gluconeogenesis could affect precursor availability for overproduction of capsular polysaccharides (Lee et al., 2016; Wu et al., 2024; Yeow et al., 2023). This finding underscores the possible role of non-capsular genes in conferring the hypermucoid phenotype and underscores the need for further research on these genes.

A significantly enhanced siderophore production is another critical factor distinguishing hvKp from cKp. Quantitative analysis showed a 6-to-10-fold increase in siderophore production compared to cKp strains, particularly aerobactin (Russo et al., 2014). The key siderophores and their role in virulence are discussed in Table 2. Aerobactin production and transport are controlled by the genes *iucABCD* and *iutA,* respectively, with *iucB* acting as a crucial link in the biological activity of aerobactin. Russo et al. (2014, 2015) showed that more than 90% of total siderophore activity in hvKp is aerobactin-dependent, and deletion of the *iucA* gene significantly reduced bacterial survival in human ascites and serum, as well as virulence in murine models. Furthermore, Liu et al., 2022 also confirmed that *iucB*, a key gene in the aerobactin biosynthetic operon, significantly enhances virulence in *Galleria mellonella* larvae. Additionally, they suggested that *iucB* may suppress *rmpA* expression, underscoring its independent dominance in virulence control.

**Table 2:** Key siderophores of hvKp, their genetic control, host interaction, and role in virulence.

Siderophore	Genetic control	Host interaction/Immune evasion	Virulence impact	References
Aerobactin	Encoded by *iucABCD* on virulence plasmid; often co-localized with *rmpA*	Not bound by lipocalin-2, not affected by low pH, enhances immune evasion	Considered the most important virulence factor in hvKp; strongly enhances growth in iron-poor environments	Liu et al. (2022) and Yeh et al. (2007)
Yersiniabactin	Encoded in the *ybt* locus in the chromosome with the ICEKp element; regulated by mobile elements	Some immune evasion, sensitive to oxidative stress and acidic pH, modulates the host stress response	Contributes to virulence; associated with serum survival, tissue dissemination	Abbas et al. (2024) and Hsieh et al. (2008)
Salmochelin	Found in the *iroA* locus on virulence plasmids; often near *iuc* genes	Modified form of enterobactin, which avoids binding by lipocalin-2, enhances immune evasion	Enhances survival in the host by bypassing lipocalin-2-mediated restriction	Fischbach et al. (2006)
Enterobactin	Core chromosomal siderophore; encoded by the *entABCDEF* genes	Sequestered by lipocalin-2, ineffective in the host iron competition	Primary siderophore in cKp; redundant in hvKp unless modified to salmochelin	Abbas et al. (2024)

An appropriate reason for the preferential expression of aerobactin in hvKp remains unknown. The biosynthetic and functional efficiency might be a probable reason for its increased expression. According to Bailey et al. (2018), *iucABCD-iutA* encodes a stereospecific biosynthetic pathway that is both structurally and enzymatically optimized for efficient aerobactin synthesis. Besides, its recycling capability and lipocalin-2-mediated efficiency in transferring iron from transferrin without any interference from albumin or immunoglobulin may enhance its biological activity. Fe^3+^-aerobactin is very stable under acidic conditions, facilitating its prevalence at infection sites that often have lower local pH (Abbas et al., 2024). Also, its expression significantly increases in iron-limited conditions through *fur* derepression (Hsieh et al., 2008). It also has functions that extend beyond iron scavenging, such as modulating capsule production or metabolic adaptation in nutrient-depleted tissues, suggesting complex regulatory networks between different virulence systems (Russo et al., 2014). Therefore, aerobactin serves as a prominent anti-virulence target due to its biosynthetic efficiency, its unique resistance to host immunity, and regulation of other plasmid-encoded virulence genes.

A defining characteristic of hypervirulent *K. pneumoniae* (hvKp) is a large pLVPK-like virulence plasmid carrying multiple virulence loci. For the stable maintenance of this virulence plasmid within hvKp populations, which is essential for hypervirulence, the toxin-antitoxin (TA) system plays a significant role. The RES-Xre TA system (*knaAT* locus), directly supports hvKp virulence by stabilizing the large virulence plasmid often found in this pathogen (Chen et al., 2024). This plasmid carries critical virulence genes responsible for hypermucoviscosity and siderophore production. The toxin KnaT and its cognate antitoxin KnaA function together as a classical type II TA pair, where KnaT inhibits bacterial growth unless neutralized by KnaA. Disruption of this system results in the gradual loss of the virulence plasmid and, consequently, reduced virulence in animal models. Thus, this TA system serves as a genetic safeguard, ensuring the stable inheritance of virulence determinants and supporting hvKp’s pathogenicity and host survival.

## Host microenvironment and virulence modulation

Hypercapsule production, including the other encoded virulence factors pivotal to hvKp pathogenicity, contributes differently to the pathogen’s success at different infection sites, where the microenvironment and nutrient availability can vary. Recent studies have underlined the critical role of host-derived nutrients and microenvironmental cues in regulating the virulence and colonization capabilities *K. pneumoniae*, particularly hypervirulent strains (Cheng et al., 2022; Jousset et al., 2018; Zaborskytė et al., 2025). However, these factors have been identified in individual assays rather than in a competitive context. For example, host-derived metabolites like arginine have been shown to influence hypermucoviscosity in hvKp KPPR1 strain via arginine response regulator ArgR, while L-fucose influences gut colonization in mice by modulating the *rmp* regulatory network without affecting the total capsular polysachharide levels in hvKp strain KPPR1 and mice gut, respectively (Hudson et al., 2022; Ryan et al., 2025). Also, nutrient-deficient and antimicrobial-rich environment inhibits hvKp mucoidy by downregulating *rmpADC*. However, this inhibition can be circumvented by spontaneous mutations in the *wzc* gene, which encodes a tyrosine autokinase implicated in CPS export. Thus, the *wzc* mutations reinstate hypermucoviscosity despite being suppressed in nutrient-deficient environment, demonstrating hvKp’s adaptive ability for ‘stealth virulence’ even in adverse conditions (Ernst et al., 2020; Zaborskytė et al., 2025; Hu et al., 2024; Khadka et al., 2023). Further, this environmental sensing on hvKp virulence is illustrated by another study where a two-component response regulator OmpR has been found to attenuate hypemucoviscosity by repressing *atp* operon (F-type ATP synthase) and *gcvTHP* operon (glycine cleavage system) (Wang L. et al., 2023).

However, these phenotypic outcomes might vary across studies due to differences in strain background and experimental conditions, underscoring the need for further studies to broaden their relevance across different strains sourced from both clinical and environmental settings, which can help determine targetable nodes to combat hvKp pathogenesis.

## Quorum sensing systems in virulence regulation

Quorum sensing (QS) significantly affects hvKp virulence by regulating the expression of essential pathogenicity factors (Chen et al., 2020; Zhou et al., 2024). The LuxS/AI-2 quorum sensing system, an important communication mechanism in bacteria, intricately regulates hvKp pathogenicity, with its influence often dependent on the strain’s unique mucoid phenotype and the bacterial developmental phase (Santajit and Sookrung, 2022; Shafaie et al., 2025; Tang et al., 2024). LuxS inhibits capsule development in hvKp during the early growth phase, when cell density is low, while facilitating capsule and biofilm formation and enhancing overall pathogenicity as the bacterial population attains higher densities in the late growth phase (Balestrino et al., 2005; Tang et al., 2024). However, the effect of LuxS on the mucoid/non-mucoid phenotype of cKp, as well as on biofilm production, is the same across growth phases, a significant distinguishing feature between these two pathotypes (Chen et al., 2020; De Araujo et al., 2010; Tang et al., 2024).

The *QseBC* two-component QS system has also been shown to influence hvKp pathogenicity. Studies demonstrate that the lack of *QseC*, an essential element of this system, results in overexpression of genes associated with biofilm formation, the bacterial type VI secretion system, and siderophore production, augmented resistance to serum-mediated killing, all of which are recognized virulence factors in hvKp (Gong et al., 2025; Khunsri et al., 2023; Lv et al., 2022). *SdiA*, an orphan LuxR-type regulator in hvKp, functions primarily as a repressor of biofilm formation and fimbriae production, which are crucial for bacterial adherence (Pacheco et al., 2021; Patankar and González, 2009; Santajit and Sookrung, 2022). The complex, interrelated functions of these QS systems highlight the advanced regulatory mechanisms that control hvKp pathogenicity. Additional investigation into these pathways is expected to uncover new targets for therapeutic strategies designed to mitigate the virulence of this increasingly alarming disease.

## Transmission of the virulence plasmid

The evolution of hvKp from cKp and, more importantly, the emergence of carbapenem-resistant hvKp (CR-hvKp) have garnered significant attention, as they are associated with higher morbidity and mortality due to their simultaneous immune evasion and antimicrobial resistance. The continuous evolution and exchange of plasmids harboring virulence and carbapenem-resistance determinants lead to the formation of hvKp and CR-hvKp from cKp and CR-cKp, respectively.

Although virulence plasmids of hvKp, like the prototypical pLVPK, have generally been regarded as non-conjugative due to the lack of a complete *tra* operon, they can become mobilizable by interaction with co-resident conjugative plasmids. Xu et al. (2021) investigated the mechanisms underlying such mobilization. Using ST11 carbapenem-resistant *Klebsiella pneumoniae* (CRKP) as a model, four distinct mechanisms of transmission for the non-conjugative virulence plasmid were identified, *viz.*, direct transfer, co-transfer with an IncF plasmid, homologous recombination, and double single-strand exchange at specific 28-bp fusion sites. Their *in-silico* investigation indicated that 98.8% of virulence plasmids contained these fusion sites, and all carried an *oriT* site, suggesting widespread potential for mobilization via fusion with helper plasmids. Zhao et al. (2021) reported the formation of a hybrid plasmid via the fusion of a non-conjugative pLVPK-like virulence plasmid with a blaKPC-2-harboring conjugative plasmid, resulting in the co-transfer of virulence and resistance traits in an ST592 hvKp strain.

Conjugative virulence plasmids that are globally transmitted have further complicated the situation. A conjugative plasmid p16HN200-Vir, which contains both the *iucABCD-iutA* (aerobactin siderophore operon) and carbapenemase genes such as *bla_NDM-1_* or *bla_OXA-48,_* has been documented by Xu et al. (2025). This plasmid was detected in 19 sequence types (STs), with prevalence in China and the United Kingdom, indicating global spread. Another novel hybrid conjugative virulence plasmid, pAP855, in the ST23 hvKp strain AP8555 has been reported by Fan et al. (2022). This plasmid harbors an origin of transfer (oriT), a complete type IV secretion system comprising multiple *tra* and *trb* genes, and a CcdAB type II toxin-antitoxin system that maintains plasmid stability. This allows for high transmissibility, thereby increasing its epidemiological potential.

Vesicle-mediated horizontal gene transfer has emerged as another unique and effective method of gene spread. HvKp-produced outer membrane vesicles (OMVs) may encapsulate and transfer virulence plasmids to other *K. pneumoniae* strains and *E. coli* (Hua et al., 2022; Li et al., 2022). The recipient strains showed increased expression of virulence factors and, in some cases, converted ESBL-producing cKp bacteria into extremely virulent, multidrug-resistant phenotypes capable of inducing severe infections in mouse models. This type of horizontal gene transfer is especially challenging in clinical and environmental reservoirs.

The gene plasticity of *K. pneumoniae* has also been reported to be enhanced by the mobile genetic elements (MGEs). According to Han et al. (2025), MGEs such as IS26 and Tn3 typically flank virulence and resistance genes and can facilitate their transposition across plasmids or the chromosome. These components frequently function as genetic scaffolds, allowing the development of complex plasmids containing both characteristics, which can then be horizontally transferred by conjugation or vesiculation.

Thus, the transition of hvKp to CR-hvKp is not unidirectional. It can occur by acquiring resistance plasmids via hvKp, acquiring virulence plasmids via CRKP, or acquiring fusion or hybrid plasmids via classical *K. pneumoniae* (cKp) strains. Such convergence events are becoming more common, particularly under selection pressure from antibiotic use, which contributes to hospital outbreaks and the global spread of CR-hvKp.

These studies emphasize the development and evolution of hvKp via genetic rearrangement through sequence insertion, conjugation, plasmid fusion, helper-mediated mobilization, and OMV-based vesiduction. Also, the structural and genetic plasticity in hvKp may facilitate the spread of cointegrated virulence plasmids, despite the associated fitness cost. This structural and genetic fluidity presents significant challenges for clinical management and infection control, emphasizing the urgent need for global surveillance.

## Antibiotic resistance in hvKp

Hypervirulent *Klebsiella pneumoniae* was first detected in the Asia-Pacific region, specifically in Taiwan (Liu et al., 1986). Since its emergence, hvKp has spread worldwide, causing illnesses across multiple continents. The hvKp strains were initially susceptible to antibiotics. However, with time, these strains have acquired antibiotic resistance genes, forming MDR-hvKp. This appears to occur more readily through the transformation of cKp strains, which are known for their robust gene uptake ability.

The global emergence of carbapenem resistance in hvKp has posed a serious threat to public health. It has been reported that CR-hvKp is being formed as a result of the acquisition of virulence plasmids by specific high-risk MDR clones, such as ST11, ST15, ST147, CG11, CG43, and ST23 (Kulkarni et al., 2024). ST23 hvKp isolates, initially susceptible to antimicrobials, have also evolved into MDR-hvKp. China is a major endemic area for CR-hvKp, with blaKPC-2 predominating in Chinese hospitals, promoting the emergence of hv-CRKp and CR-hvKp strains (Pu et al., 2023). Also, in early 2024, the Global Antimicrobial Resistance and Surveillance System on Emerging Antimicrobial Resistance Reporting (GLASS-EAR) reported a significant rise in the detection of ST23, particularly those harboring carbapenem resistance genes (Antimicrobial Resistance, Hypervirulent *Klebsiella pneumoniae* - Global Situation, 2024). Also, a study conducted in India characterized the *catA1* gene on the virulence plasmid of ST23 MDR-hvKp isolates, emphasizing its direct insertion into the virulence plasmid, which confers chloramphenicol resistance (Shankar et al., 2020). This lineage has persisted for several years and has been documented in at least one nation across all six World Health Organization (WHO) regions. There is an increasing trend in the carbapenem resistance pattern since their emergence, with variations in the prevalence of carbapenemase gene variants (Beig et al., 2024).

**Figure 4:**
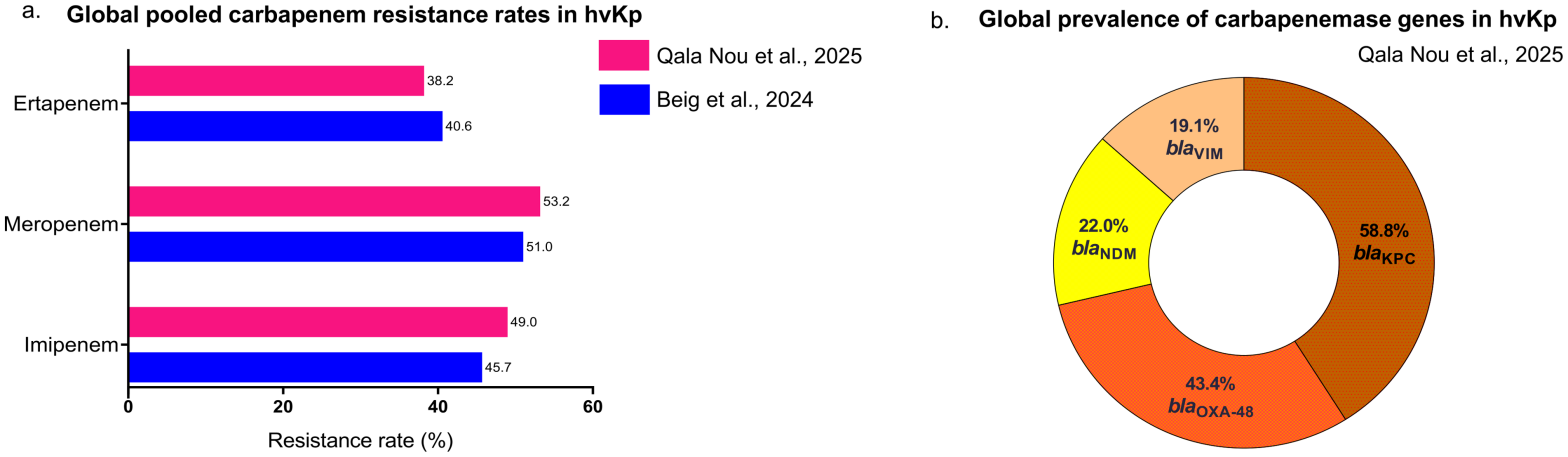
Global trends in carbapenem resistance and carbapenemase distribution among hypervirulent *Klebsiella pneumoniae* (hvkp) isolates. **(a)** Global pooled resistance rates for the carbapenems imipenem, meropenem, and ertapenem, replotted from the meta-analyses of Beig et al. (2024) and Qala Nou et al. (2025). **(b)** Global prevalence of carbapenemase gene variants among hvKp isolates based on the meta-analysis of Qala Nou et al. (2025). The pie chart illustrates the distribution of major carbapenemase types worldwide. These data underscore the predominance of KPC-type carbapenemases in hvKp and the widespread global dissemination of diverse carbapenemase determinants contributing to carbapenem resistance.

The global resistance rates to carbapenems and the prevalence of carbapenemase genes in hvKp are shown in Figure 4. The resistance rates for the carbapenems imipenem, meropenem, and ertapenem were replotted from the datasets reported by Beig et al. (2024) and Qala Nou et al. (2025) (Figure 4a). The prevalence of carbapenemase gene variants was derived from the meta-analysis of Qala Nou et al. (2025) (Figure 4b). The pooled analyses demonstrated alarmingly high global resistance rates among hvKp isolates against carbapenem antibiotics. Beig et al. (2024), analyzing 77 studies across 14 countries published between 2014 and 2023, reported resistance rates of 45.7% for imipenem, 51.0% for meropenem, and 40.6% for ertapenem. Similarly, Qala Nou et al. (2025), based on 36 studies encompassing 1,098 hvKp isolates published between 2014 and 2024, reported pooled resistance rates of 49.0% for imipenem, 53.2% for meropenem, and 38.2% for ertapenem. Analysis of carbapenemase determinants further revealed bla_KPC_ as the most prevalent carbapenemase gene (58.8%), followed by bla_OXA-48_ (43.4%), bla_NDM_ (22.0%), and bla_VIM_ (19.1%) (Qala Nou et al., 2025). Collectively, these meta-analytic data were used to illustrate the global burden and recent trends in carbapenem resistance among hvKp isolates. To treat the CR-hvKp infection, colistin was used as a last resort, but reports of colistin-resistant CR-hvKp have already been documented (Jian et al., 2024; Zhang et al., 2021).

Reports from India have increasingly emphasized the prevalence of hvKp with resistance to prescribed treatment options, especially in nosocomial environments. The resistance rates for CR-hvKp vary significantly, ranging from 4.4 to 100% with mortality rates ranging from 30 to 60% (Koli et al., 2025). The reason for carbapenem resistance is primarily attributed to the acquisition of New Delhi Metallo-*β*-lactamase (*bla*_NDM_) and *bla*_OXA-48_ (Banerjee et al., 2021; Khan et al., 2017; Rafiq et al., 2016; Raj et al., 2022; Yadav et al., 2023). However, a study also reported the occurrence of colistin resistance in hvKp sourced from neonatal sepsis in India (Banerjee et al., 2021). The worldwide proliferation of drug-resistant hvKp indicates that this issue transcends local boundaries and constitutes a significant global health hazard, necessitating coordinated international action.

## Management of hvKp infection

### Conventional antibiotic therapy

Effective management of hvKp infection through antibiotic therapy requires a multifaceted approach, including combination therapy, particularly given the rising cases of MDR-hvKp. Empiric treatment options for hvKp infections differ based on the site of infection. In case of liver abscesses, pneumonia, and other intra-abdominal infections, recommended antibiotic regimens include β-lactam/β-lactamase inhibitor combinations, third-generation cephalosporins, fluoroquinolones, carbapenems or aminoglycosides. Carbapenems are preferred in central nervous system infections due to their penetration into cerebrospinal fluid. For endophthalmitis, intravitreal administration of antibiotics such as cefazolin, ceftazidime, aminoglycosides, or imipenem is typically coupled with intravenous antibiotics, mostly cephalosporins, to achieve both local and systemic control (Bennett et al., 2019; Chou and Kou, 1996; Russo and Marr, 2019; Siu et al., 2012). These empirical strategies must be tailored to local resistance profiles to ensure optimal outcomes in hvKp.

However, multidrug-resistant hvKp is becoming more common, which may be linked to the rise in healthcare-associated infections. The rising prevalence of multi- or extensive antibiotic resistance exacerbates the present treatment regimes, making traditional antimicrobial therapies progressively ineffective. The growing challenge of hvKp antibiotic resistance has driven the exploration of antivirulence strategies as alternative or adjunctive therapies that disarm pathogens rather than directly targeting their survival.

### Antivirulence strategies

**Figure 5:**
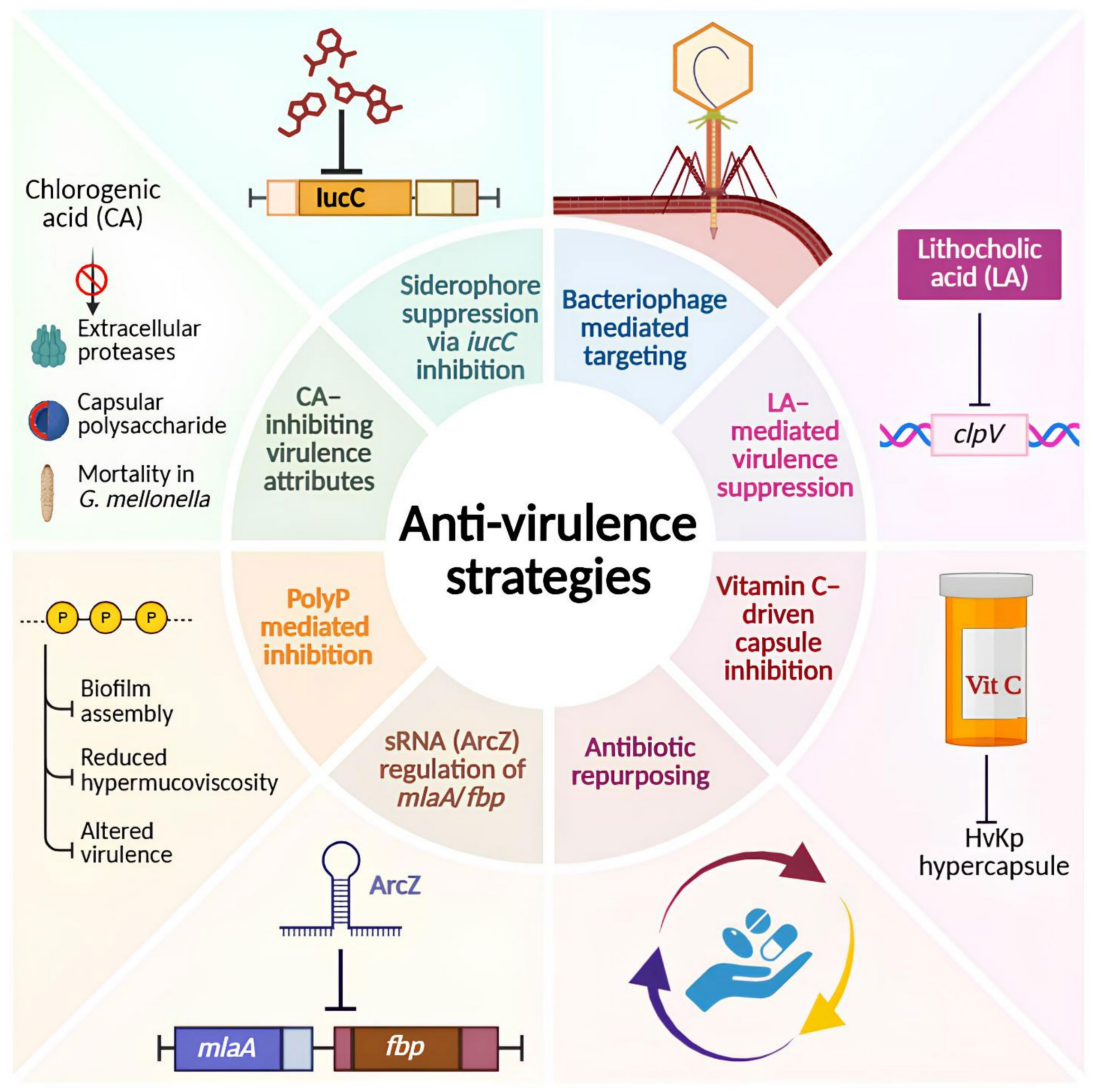
Schematic representation of emerging anti-virulence strategies targeting hypervirulent *Klebsiella pneumoniae* (hvKp). Strategies include: chlorogenic acid reducing extracellular proteases, capsular polysaccharide production, and *Galleria mellonella* mortality; inhibition of the siderophore synthesis gene *iucC*; bacteriophage therapy; lithocholic acid downregulating the type VI secretion system gene *clpV*; vitamin C as a potential antivirulence agent; regulation of *mlaA* and *fbp* through ArcZ; and targeting polyP metabolism to prevent gut colonization and reduce virulence in hvKp. These strategies aim to attenuate pathogenicity while minimizing selective pressure for antibiotic resistance. Created with BioRender.com.

Antivirulence therapy serves as a promising therapeutic paradigm that selectively disarms the pathogen. Several approaches have been undertaken to target the pathogenicity of hvKp, which includes targeting the capsular polysaccharides, siderophores, as well as the type VI secretion system of hvKp, which will be discussed in this section (Figure 5). Also, the repurposing of antimicrobials at subinhibitory concentrations has been explored.

Aerobactin, a critical virulence factor of hvKp, has been an attractive target for the development of small-molecule inhibitors. Aerobactin synthetase IucC has been targeted for high-throughput biochemical screening to identify inhibitors. Several inhibitors with micromolar IC_50_ values have been found in initial screening; however, more optimization is needed to find molecules appropriate for preclinical research (Russo and Gulick, 2019). This represents an important step in targeting virulence mechanisms independent of traditional resistance pathways.

An alternative approach involves attenuating hvKp virulence determinants using a sub-inhibitory dose of antibiotics. This approach involves repurposing existing drugs that adhere to the core principle of diminishing the virulence while maintaining the selective pressure sufficiently low to delay the evolution of antimicrobial resistance. Sub-MIC levofloxacin has been shown to induce capsule thinning, reduced capsular polysaccharide production, and decreased mucoviscosity. Levofloxacin also suppressed the expression of siderophore production genes *iroN* and *iucA*, rendering hvKp susceptible to the host immune system (Ma et al., 2022). Although, *K. pneumoniae* shows intrinsic resistance to rifampicin, its antimucoviscous activity is pronounced. Additionally, a reduction in capsule thickness at a sub-inhibitory concentration against hvKp, without affecting siderophore production, suggests a selective antivirulence mechanism focused on capsule suppression. Sub-MIC of rifampicin suppresses the transcriptional response of *rmpA* and its downstream capsule biosynthesis genes, including *magA*, *galF*, *wzi*, and *manC*. These unique activities of levofloxacin and rifampicin position them as a potential adjunctive therapy to enhance the efficacy of bactericidal antibiotics (Namikawa et al., 2019).

Vitamin C combines bactericidal, anti-biofilm, and antivirulence capabilities with a complex action on carbapenem-resistant hvKp (CR-hvKp). Vitamin C takes advantage of the iron-rich environment characteristic of hvKp at greater concentrations to produce reactive oxygen species (ROS). By inhibiting exopolysaccharide (EPS) synthesis and impairing efflux pump performance, vitamin C reduces surface deposition of both EPS and CPS at sub-MICs, hence inhibiting biofilm formation. Several virulence genes, including *rmpA*, *rmpA2*, *fimB*, *mrkJ*, *ecpA*, *luxS*, and *galF* were downregulated, according to transcriptional studies. This led to attenuated pathogenicity, with reduced capsule and biofilm development. Moreover, CR-hvKp did not show resistance to vitamin C following serial passages and dramatically increased survival in mouse models, providing clinical benefits such as safety, cost, and low toxicity, and highlighting its promise as a treatment option (Xu et al., 2022).

Chlorogenic acid (CA), a plant-derived phenolic compound, has been demonstrated to act as an antivirulence agent against hvKp. At sub-inhibitory concentrations, CA attenuates capsule formation, protease activity, and biofilm formation without significantly affecting bacterial growth, primarily by inhibiting AI-2 signaling and downregulating QS-associated genes, including *luxS* and *mrkA*. The absence of a significant effect on bacterial growth highlights its promising potential as an antivirulence strategy against hvKp, as it targets pathogenicity rather than bacterial viability (Wang et al., 2022).

Inhibiting bacterial secretion systems, especially the Type VI secretion system (T6SS), constitutes another antivirulence approach. The *clpV* gene, crucial for T6SS functionality and reassembly, plays a significant role in hvKp pathogenicity. The ablation of *clpV* markedly diminished mucoid phenotype expression, biofilm development, and pathogenicity in *G. mellonella* infection models, without affecting bacterial proliferation. Transcriptomic study of *clpV*-deleted hvKp revealed extensive reduction of virulence factors, including *iucA*, *iroB*, *entA*, *rmpA*, *peg-344*, and *fimA*. Lithocholic acid has been recognized as a possible *clpV* inhibitor, diminishing its expression in a dose-dependent manner and reducing hvKp pathogenicity *in vitro* (Liu et al., 2025). This approach of targeting components of the bacterial secretion system, such as *ClpV*, is a promising antivirulence strategy with a lower likelihood of resistance emergence.

Another strategy targets inorganic polyphosphate (polyP) metabolism by inhibiting polyphosphate kinase 1 (PPK1), the primary enzyme responsible for polyP synthesis in *K. pneumoniae*. In hypervirulent ST23 strains, the disruption of *ppk1* significantly reduces capsule production, modifies capsule morphology, mitigates hypermucoviscosity, and impairs biofilm biomass, extracellular polymeric substance production, and biofilm structural organization. Proteomic profiling demonstrated the downregulation of capsule synthesis proteins, type III fimbriae components (MrkC, MrkD, MrkH), and colibactin biosynthetic enzymes, as well as siderophore dysregulation. By reducing the selective pressure for resistance development, PPK1 inhibition provides a promising approach to disarm hvKp by impairing multiple virulence determinants without affecting bacterial viability (Rojas et al., 2024).

Bacteriophage therapy is advancing as a potent alternative to antibiotics, particularly for multidrug-resistant infections such as CR-hvKp. Phages employ lytic processes and depolymerase enzymes that degrade capsular polysaccharides, increasing bacterial vulnerability to immune clearance. A novel phage, PCCM_KpP1172, exhibits strong host specificity for CR-hvKp ST11-KL64 strains and lacks virulence or antibiotic resistance genes, making it an attractive therapeutic option. *In vivo* investigations in *G. mellonella* have demonstrated substantial survival advantages after phage therapy (Yinghan et al., 2024). Thus, this genetically safe, highly specific bacteriophage can be a precision tool against multidrug-resistant hvKp infections.

Recent findings also underscore the regulatory role of small RNAs (sRNAs) in modulating hvKp virulence by post-transcriptionally suppressing the hypermucoviscosity-associated genes *mlaA* and *fbp*. ArcZ functions through direct RNA–RNA interactions with the 5’ UTRs of these mRNAs, inhibiting their translation (Dubois et al., 2024; Wu et al., 2024). Overexpression of ArcZ reduces bacterial burden in mice and leads to a drastic reduction in capsule retention, virulence, and hypermucoid phenotype in hypervirulent and carbapenem resistant clinical strains of *K. pneumoniae* (Dubois et al., 2024; Wu et al., 2024). These findings suggest that ArcZ and its targets may represent therapeutic entry points for RNA-based inhibition strategies.

**Figure 6:**
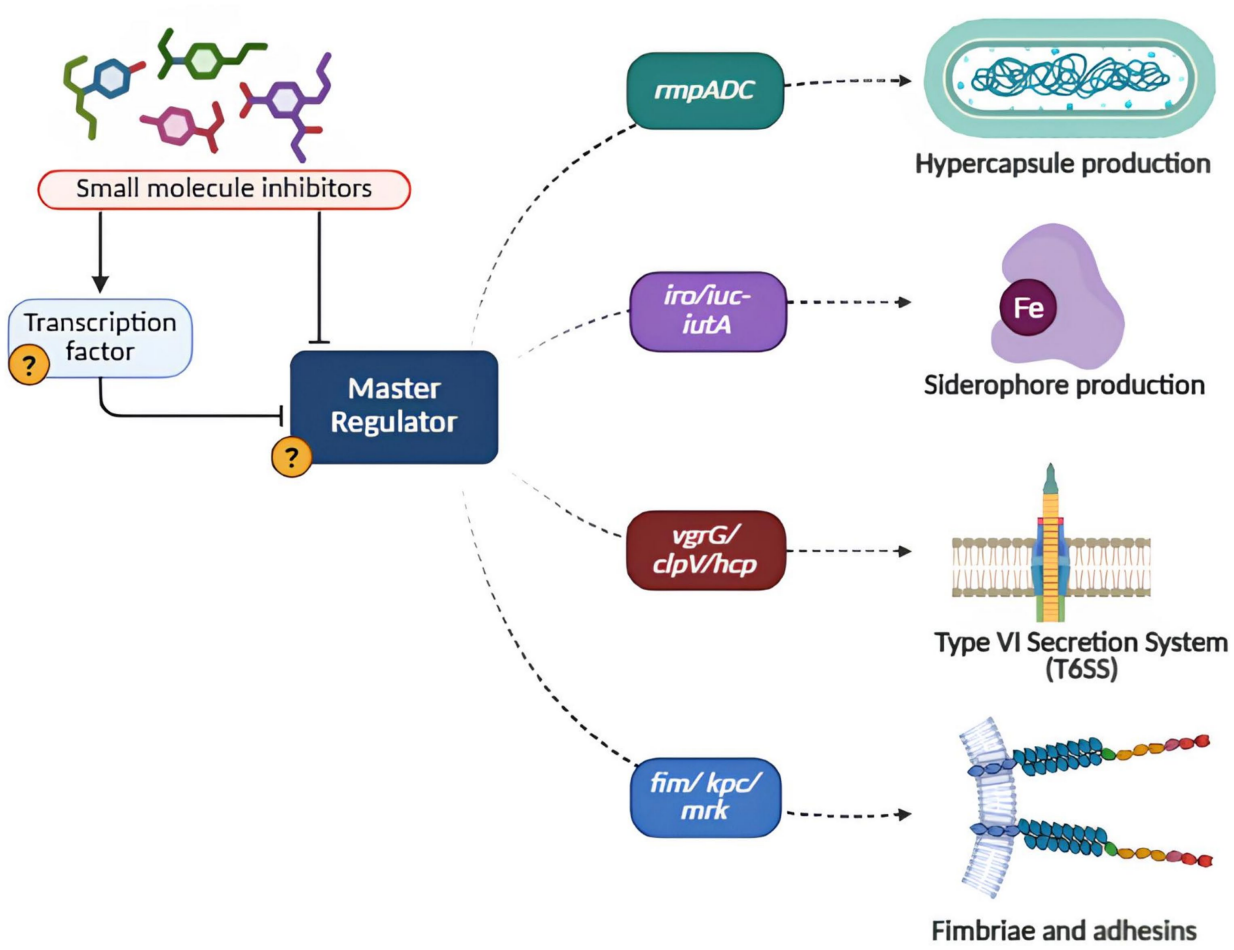
Hypothetical model of a master regulator coordinating virulence in hypervirulent *Klebsiella pneumoniae*. The putative master regulatory node integrates upstream signals, potentially through transcription factors, and can be targeted by small-molecule inhibitors. This central regulator is proposed to control key virulence-associated loci, including *rmpADC* (hypercapsule production), *iro/iuc-iutA* (siderophore biosynthesis), *clpV* (type VI secretion system, T6SS), and *fim/mrk* (fimbriae and adhesins). By targeting the regulator or its transcriptional inputs, it may be possible to simultaneously attenuate multiple virulence pathways and identify novel antivirulence therapeutic strategies. Created with BioRender.com.

The overall virulence in hvKp is orchestrated by a complex network of genes. Identifying the regulatory circuit and its master regulator could offer a promising therapeutic target for developing small-molecule inhibitors of that regulator (Figure 6).

## Conclusion and future direction

Despite major advances in tracking hvKp virulence, several key gaps, if addressed, would improve prevention, diagnosis, and treatment of hvKp. First, a standardized multi-omic panel incorporating genotypes, epigenetic markers, niche-linked expression of virulence factors, and quantitative siderophore output should be prospectively validated against patient outcomes and compared head-to-head with the string test. A rapid assay kit that reports ‘hvKp probability score’ is the need of the hour. Network models that integrate QS with plasmid-encoded virulence regulators may point to a tractable master regulator that can prioritize druggable nodes and their targets. Implementation of one health programs to evaluate the stewardship and infection control bundles that specifically target the metastatic phenotype of hvKp and environmental persistence is another promising approach for controlling the hvKp infection and its spread. Furthermore, prospective clinical trials exclusively targeting hvKp infections, capturing endpoints like metastatic complications, relapse rates, and resistance emergence, are urgently needed to bridge the gap between scientific interventions and real-time patient outcomes.

Thus, to tackle this pathogen with flexible genetic architecture, aligning mechanistic insights with translational pipelines and fit-for-purpose clinical trials can blunt hvKp’s metastatic potential while slowing resistance, shifting patient outcomes from high-morbidity invasive disease to preventable, containable infection.
